# Supplementary material for: FERN – a Java framework for stochastic simulation and evaluation of reaction networks
Source: BMC Bioinformatics. 2008 Aug 29;9:356. doi: 10.1186/1471-2105-9-356 (PMC2553347; doi:10.1186/1471-2105-9-356)
Supplement: Additional file 1 — FERN distribution, Version 1.3. This archive contains the FERN source code and binaries as well as documentation and example models in FernML and SBML. [file 1471-2105-9-356-S1.zip › fern/doc/javadoc/fern/simulation/observer/package-summary.html]

fern.simulation.observer


---


|  |  |  |
| --- | --- | --- |
| **Overview** \| **Package** \| Class \| **Use** \| **Tree** \| **Deprecated** \| **Index** \| **Help** | |  |
| **PREV PACKAGE**   **NEXT PACKAGE** | **FRAMES**    **NO FRAMES**     **All Classes** |  |


---

## Package fern.simulation.observer

Provides classes to observe certain aspects of simulations.

**See:**
  
          **Description**

| **Interface Summary** | |
| --- | --- |
| **GnuPlotObserver** | Interface for certain observer which can produce gnuplot data. |

| **Class Summary** | |
| --- | --- |
| **AmountAtMomentObserver** | Observes the amount of some molecule species at a certain moment. |
| **AmountIntervalObserver** | Observes amounts of molecule species repeatedly after certain intervals. |
| **FireTypeObserver** | Observes the different types of firings. |
| **InstantOutputObserver** | Simply prints out every event that the simulator reports to the observer. |
| **IntervalObserver** | Base class for observing certain aspects repeatedly after a given interval. |
| **LeapObserver** | Observes the amount of given molecule species at each `step`. |
| **MultiAmountIntervalObserver** | Observes amounts of molecule species repeatedly after certain intervals. |
| **Observer** | Implementing classes are used to observe certain aspects of simulations. |
| **RandomNumberGeneratorCallObserver** | Observes the calls to the random number generator and count the number of drawn random number of different distributions. |
| **ReactionIntervalObserver** | Observes number of firings of reactions repeatedly after certain intervals. |
| **TriggerObserver** |  |

## Package fern.simulation.observer Description

Provides classes to observe certain aspects of simulations.

---


|  |  |  |
| --- | --- | --- |
| **Overview** \| **Package** \| Class \| **Use** \| **Tree** \| **Deprecated** \| **Index** \| **Help** | |  |
| **PREV PACKAGE**   **NEXT PACKAGE** | **FRAMES**    **NO FRAMES**     **All Classes** |  |


---
